# New feature: themed sections

**DOI:** 10.1111/eva.12070

**Published:** 2013-03-28

We are pleased to introduce ‘Themed Sections’, as a new feature in *Evolutionary Applications* that we will be publishing two or three times a year, each comprising three to five papers that can be either original empirical work, meta-alysis or synthesis. The first of this series was led by guest editors Ananias Escalante and Sudir Kumar and comprises three papers in the emerging interdisciplinary field Phylomedicine. In a comparative genomics framework, Teeling et al. (2013) investigated ‘deafness’ genes across 69 divergent species of mammals, looking for adaptive evolution, as well as regions associated with disease mutations. Then, Tkaczuk et al. (2013) used state-of-the-art crystallography approaches to resolve the crystal structures of two universal stress proteins (USP) from *Archaeoglobus fulgidus* and *Nitrosomonas europaea*. This work is the first complete synthesis of the structural properties of 26 universal stress proteins available in the Protein Data Bank. Finally, Gerek et al. (2013) propose a novel way of quantifying the dynamic properties of individual residues in any protein which they applied to assess the dynamics of 100 human proteins some of which bear critical positions in disease-associated population variations.

Papers published in Themes sections will receive the same rigorous, double-blind, high-standard peer review as all of our papers, and will be prioritised through the rest of the publication process. The topics to be covered will be mainly commissioned by us on the basis of their relevance and timeliness to the general field of applied evolution. However, we also encourage you to contact the Editor-in-chief for suggestion of a themed section that you feel particularly timely and that you would be willing to lead as guest editor.

